# The relationship between maximum tolerance and motor activation during transcutaneous spinal stimulation is unaffected by the carrier frequency or vibration

**DOI:** 10.14814/phy2.14397

**Published:** 2020-03-13

**Authors:** Gerome A. Manson, Jonathan S. Calvert, Jeremiah Ling, Boranai Tychhon, Amir Ali, Dimitry G. Sayenko

**Affiliations:** ^1^ Department of Neurosurgery Center for Neuroregeneration Houston Methodist Research Institute Houston TX USA; ^2^ Mayo Clinic Graduate School of Biomedical Sciences Mayo Clinic Rochester MN USA

**Keywords:** carrier frequency, pain tolerance, Russian current, transcutaneous spinal stimulation, vibration

## Abstract

Transcutaneous spinal stimulation (TSS) is a useful tool to modulate spinal sensorimotor circuits and has emerged as a potential treatment for motor disorders in neurologically impaired populations. One major limitation of TSS is the discomfort associated with high levels of stimulation during the experimental procedure. The objective of this study was to examine if the discomfort caused by TSS can be alleviated using different stimulation paradigms in a neurologically intact population. Tolerance to TSS delivered using conventional biphasic balanced rectangular pulses was compared to two alternative stimulation paradigms: a 5 kHz carrier frequency and biphasic balanced rectangular pulses combined with vibrotactile stimulation. In ten healthy participants, tolerance to TSS was examined using both single‐pulse (0.2 Hz) and continuous (30 Hz) stimulation protocols. In both the single‐pulse and continuous stimulation protocols, participants tolerated significantly higher levels of stimulation with the carrier frequency paradigm compared to the other stimulation paradigms. However, when the maximum tolerable stimulation intensity of each stimulation paradigm was normalized to the intensity required to evoke a lower limb muscle response, there were no statistical differences between the stimulation paradigms. Our results suggest that, when considering the intensity of stimulation required to obtain spinally evoked motor potentials, neither alternative stimulation paradigm is more effective at reducing discomfort than the conventional, unmodulated pulse configuration.

## Graphical abstract

Studies using transcutaneous spinal stimulation (TSS) have argued that using a burst‐modulated kHz frequency (carrier frequency) to administer spinal stimulation is more optimal because higher stimulation intensities can be tolerated as compared to pulsed stimulation. The present study sought to test whether methods used to reduce discomfort in neuromuscular stimulation are applicable to TSS. To this end, we compared tolerance to TSS administered with pulsed‐current stimulation to two methods found to reduce pain in neuromuscular stimulation: the carrier frequency, and pulsed current stimulation with the addition of local vibration. In sum, the results indicate that there are no differences between the different stimulation paradigms when maximum tolerable intensities are normalized to intensities required to elicit a spinally‐evoked motor potential.

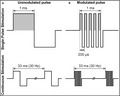

## 1 INTRODUCTION

Transcutaneous electrical spinal stimulation (TSS) is a noninvasive technique for modulating spinal neural circuits in humans (Gerasimenko et al., 2015). During TSS, stimulation is applied to the lumbosacral or cervical enlargement of the spinal cord using electrodes placed on the surface of the skin. Computational modeling and neurophysiological studies have shown that both TSS and invasive (e.g., epidural spinal stimulation) approaches primarily recruit dorsal root afferent fibers (Hofstoetter, Freundl, Binder, & Minassian, 2018; Ladenbauer, Minassian, Hofstoetter, Dimitrijevic, & Rattay, 2010; Milosevic, Masugi, Sasaki, Sayenko, & Nakazawa, 2019; Minassian et al., 2007; Sayenko et al., 2015). Additionally, many other neural structures can be directly impacted by the electrical field, including axons, synapses, neuronal cell bodies, and glial cells (Taccola, Sayenko, Gad, Gerasimenko, & Edgerton, 2018). As such, mechanisms of spinal neuromodulation may also include activation of spinal interneural networks and antidromic activation of ascending fibers in the dorsal columns. TSS has been used to increase excitability at multiple levels of the spinal neuraxis to enable motor and autonomic functions in individuals with chronic spinal cord injury (SCI) (Gad et al., 2018; Hofstoetter et al., 2013, 2015; Minassian et al., 2013, 2016; Phillips et al., 2018; Rath et al., 2018; Sayenko et al., 2019). Although TSS has been examined as a possible clinical intervention for individuals with SCI, the promising findings with regard to motor recovery and the noninvasive nature of the technique could make TSS suitable for use with other neurologically‐impaired populations. However, one of the challenges of applying TSS to other populations with preserved sensory function is the discomfort caused by electrical stimulation.

Before the stimulation arrives at the spinal cord, it must pass through multiple layers of tissues including skin, subcutaneous fat, muscles, ligaments, and vertebrae (Ladenbauer et al., 2010; Sayenko et al., 2015). The impedance of these tissues requires that high intensities of TSS be employed. High stimulation intensities can be uncomfortable for people with intact (or altered) sensation due to the activation of nociceptors in the skin beneath the electrodes (i.e., C‐fiber‐mediated pain; see Baker, Wederich, McNeal, Newsam, & Waters, 2000). Thus, a participant's ability to tolerate discomfort when exposed to TSS will determine the effectiveness of the treatment. TSS delivered using low stimulation intensities or shorter stimulation durations may not be sufficient to cause neuromodulatory effects. Therefore, the purpose of this study was to determine if tolerance to TSS can be increased using stimulation paradigms designed to reduce nociceptive pain.

Previous studies have suggested that it may be possible to reduce discomfort during TSS by using a specific waveform to deliver electrical stimulation (Gad et al., 2018; Gerasimenko et al., 2015, 2016; Rath et al., 2018; Sayenko et al., 2019). The waveform consists of 0.1–1 ms bursts of alternating biphasic rectangular pulses with a carrier frequency of up to 10 kHz. The purported advantage of the carrier frequency is that there is a decrease in the activation of cutaneous pain receptors (C‐fibers), while eliciting comparable electrophysiological responses to those induced by conventional stimulation waveforms. The proposed mechanism for the effectiveness of the kHz frequency is an extension of the temporal summation of graded potentials: It was hypothesized that the rapid depolarization and repolarization created by kHz stimulation may raise the membrane potential of larger fibers enough to cause action potentials without depolarizing unmyelinated C‐fibers (see the Gildemeister effect outlined in Ward & Chuen, 2009). Neurography studies provide some evidence for this phenomenon by showing that C‐fibers are less likely to fire in response to high‐frequency stimulation than larger fibers (see Torebjörk & Hallin, 1974, and Joseph & Butera, 2011).

Kots first proposed that using the carrier frequency paradigm for neuromuscular electrical stimulation minimized discomfort and yielded higher motor output in humans (as reported in Babkin & Timtsenko, 1977). It was reported that elite Russian athletes experienced strength gains of up to 40% following training with neuromuscular stimulation using a 2.5‐kHz alternating current applied in 10‐ms rectangular bursts at 50 Hz (known as the “Russian current” electrical stimulation; see Ward & Shkuratova, 2002). Further studies have also found that discomfort was decreased with stimulation frequencies of 0.5 kHz to 4–5 kHz but was increased at frequencies above 20 kHz (Ward, 2016; Ward & Chuen, 2009; Ward, Oliver, & Buccella, 2006; Ward, Robertson, & Ioannou, 2004). Overall, these observations suggest that the carrier frequency stimulation may be advantageous with regard to comfort (see also Moreno‐Aranda & Seireg, 1981).

Another plausible mechanism to reduce the impact of C‐fiber‐mediated pain is the application of a vibrotactile stimulus at the site of stimulation. By stimulating large Aβ fibers with a non‐noxious vibration, the transmission of pain signals by small Aδ or C‐fibers can be reduced through either primary afferent depolarization of large cutaneous fibers (see Whitehorn & Burgess, 1973 and Rudomin & Schmidt, 1999 for review), or the activation of inhibitory interneurons (see Gate Control Theory by Melzack & Wall (1965); see also Hollins, McDermott, & Harper (2014) for a review). Previous studies have found that the simultaneous application of vibrotactile stimulation (at frequencies of 50–150 Hz, see Lundeberg, Nordemar, & Ottoson, 1984) directly on, or near, the site of a painful stimulus increases pain tolerance. Vibration‐induced increases in tolerance to painful stimuli have been found during exposure to injections (e.g., Nanitsos, Vartuli, Forte, Dennison, & Peck, 2009), painful thermal stimuli (e.g., Yarnitsky, Kunin, Brik, & Sprecher, 1997), and painful electrical stimuli (e.g., Higgins, Tursky, & Schwartz, 1971). Of particular relevance to the present study, Higgins et al. (1971) found that subjective tolerance to painful electrical stimulation was higher when vibration was applied to the site of stimulation than when vibration was not applied, or when vibration was applied to a sham site (see also Bini, Cruccu, Hagbarth, Schady, & Torebjörk, 1984).

**FIGURE 1:**
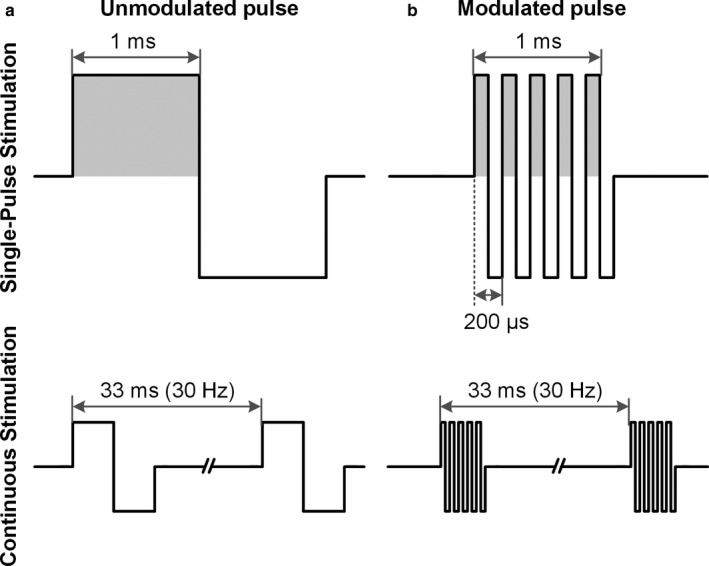
Schematics presenting (a) a single biphasic symmetric square‐wave pulse with the positive phase of 1 ms duration (pulsed‐current, unmodulated), as well as (b) burst‐modulated waveform of 1 ms duration with the carrier frequency of 5 kHz, which consisted of 5 biphasic pulses each of 200 μs duration (carrier frequency, modulated), during Single‐Pulse Stimulation (top panels) and Continuous Stimulation (bottom panels) protocols. Note the difference in the cumulative area of the positive phase (shown by the gray shaded area) during the Unmodulated and Modulated pulses

Although both the carrier frequency and the addition of surface vibration were found to be beneficial for comfort during neuromuscular stimulation, no previous study has directly compared the effectiveness of these stimulation paradigms using TSS. Thus, the objective of this study was to examine whether tolerance to TSS can be modified by altering the stimulation paradigm (see Figure 1 for a depiction of waveforms used in the present study). Three stimulation paradigms were compared: (a) unmodulated biphasic pulses (used as the baseline paradigm); (b) a 5 kHz carrier frequency; and (c) unmodulated biphasic pulses delivered with local vibration. Tolerance to each stimulation paradigm was explored using two commonly used protocols: (a) a single‐pulse threshold procedure (used for examining the efficacy of spinal sensorimotor circuitry (see Calvert, Manson, Grahn, & Sayenko, 2019; Sayenko et al., 2015); and (b) a continuous stimulation protocol at 30 Hz (used for facilitating motor functions, such as stepping in participants with SCI, see Minassian et al., 2013 and Gerasimenko et al., 2015). To gain a better understanding of the relationship between tolerance and the activation of motor pools for each stimulation paradigm, the maximum tolerable intensity was normalized to the stimulation intensity required to induce a spinally evoked motor potential (i.e., the motor threshold). Ourresults demonstrate that the relationship between tolerance and motor threshold was proportional for each stimulation paradigm, suggesting that there is no advantage of the modified stimulation paradigms for eliciting spinally evoked motor potentials as compared to the baseline condition.

## 2 MATERIALS AND METHODS

### 2.1 Participants

Ten participants took part in this experiment (2 women, age 23–30, *M* = 24 years, *SD* = 0.8; Heights: *M* = 176 cm, *SD* = 7.0; Weights: *M* = 70 kg, *SD* = 11.0). Written informed consent was obtained prior to the experiment and all procedures were approved by the Institutional Review Board at the Houston Methodist Research Institute.

### 2.2 TSS and electromyography (EMG)

Electrical stimulation was delivered using a DS8R Biphasic Constant Current Stimulator (Digitimer, UK) with a range of 0 to 1,000 mA. Stimulation was administered using two conductive self‐adhesive electrodes (PALS, Axelgaard) with a diameter of 32 mm. These electrodes served as the cathodes and were positioned on the skin laterally to the left and right of the midline and between the spinous processes of the L1 and L2 vertebrae. Two additional 50 × 100 mm self‐adhesive (PALS, Axelgaard, USA) electrodes served as the anodes and these positioned on the abdomen about 2 cm laterally left and right from the participant's midline (corresponding to about T11 to L2). The electrode positions were based on previous work where similar electrode configurations were used to activate spinal sensorimotor networks (Calvert et al., 2019; Danner et al., 2016; Hofstoetter et al., 2015). Participants laid in a prone position for the duration of the experiment.

Surface electromyogram (EMG) signals were recorded bilaterally from the lower limbs using wireless sensors (Trigno™ Avanti Delsys). The electrodes were placed longitudinally on the vastus lateralis, medial hamstrings, tibialis anterior, medial gastrocnemius, and soleus muscles. The EMG signals were differentially amplified (using a standard AC‐coupled differential amplifier with a gain of 909 and a bandpass filter of 20–450 Hz) and digitized at a sampling rate of 2,000 Hz using a PowerLab System (ADInstruments). EMG signals were synchronized to the stimulation artifact to record spinally evoked motor potentials.

### 2.3 Stimulation paradigms

Tolerance during TSS was examined using three stimulation paradigms: (a) a single biphasic symmetric rectangular‐wave pulse with the positive phase of 1 ms duration (pulsed‐current, unmodulated; see Figure 1a); (b) a burst‐modulated waveform of 1 ms duration with the carrier frequency of 5 kHz, which consisted of 5 biphasic pulses each of 200 μs duration (carrier frequency, modulated; see Figure 1b); and (c) an unmodulated pulse accompanied by 50 Hz vibration (pulsed‐current + vib). The vibration was applied over the site of stimulation (above the cathode electrodes) on the back. The amplitude of vibration was ~3 mm and it was delivered using a commercial vibrotactile stimulation device (Zhejiang Luyao Electronics Technology Co. Ltd).

### 2.4 Stimulation protocols

Two stimulation protocols were used to examine tolerance to TSS: A Single‐Pulse Stimulation protocol and a Continuous Stimulation protocol. During each protocol, participants were presented with each stimulation paradigm at an incrementally increasing stimulation intensity. In the Single‐Pulse Stimulation protocol, for the pulsed‐current and pulsed‐current + vib stimulation paradigms, the starting intensity was 10 mA and this was increased by 10 mA for each subsequent stimulation. For the carrier frequency stimulation paradigm, the starting intensity was 20 mA and this was increased by 20 mA for each subsequent stimulation. Participants received 7–10 stimulations per minute (i.e., a minimum of a 6‐s inter‐stimulus‐interval) and were given a 1‐min break after every minute of stimulation. For the pulsed‐current + vib stimulation paradigm, the vibration was delivered continuously to the stimulation site during the 1‐min stimulation period, but was turned off during the break periods. The order that the stimulation paradigms presented in the Single‐Pulse Stimulation protocol was pseudo‐randomized for each participant, and the sequences were presented in a balanced manner throughout the experiment between the participants.

Participants were instructed to verbally indicate when they had reached their maximum tolerable intensity (i.e., that they cannot tolerate another stimulation) and report the amount of pain they experienced using a numerical rating scale (NRS) from 0 to 10. A score of 0 was defined as “no pain or discomfort at all” and a score of 10 was defined as “the worst pain”. Participants received a 5‐min break before proceeding to the next stimulation paradigm.

In the Continuous Stimulation protocol, each stimulation paradigm was presented at a frequency of 30 Hz. For the pulsed‐current, and pulsed‐current + vib stimulation paradigms, 1 ms biphasic pulses were presented at a starting intensity of 5 mA and this was increased by 5 mA approximately every 5 s. For the carrier frequency stimulation paradigm, the starting intensity was 10 mA and this was increased by 10 mA every ~5 s. Participants were asked how they felt after every increase in stimulation intensity. Participants were also instructed to stop the test if they felt that they could not tolerate the stimulation for more than 30 s. In the subsequent break period, participants also reported the cause of their discomfort to the experimenter (see Discussion). The intensity at which participants stopped stimulation was recorded as the maximum tolerable intensity for the continuous stimulation protocol. The Continuous Stimulation protocol always followed the Single‐Pulse stimulation protocol and the order that the stimulation paradigms were presented in the Continuous Stimulation protocol was the same as the order used in the Single‐Pulse Stimulation protocol for a given participant.

### 2.5 Data analysis

#### 2.5.1 Electromyography (EMG)

**FIGURE 2:**
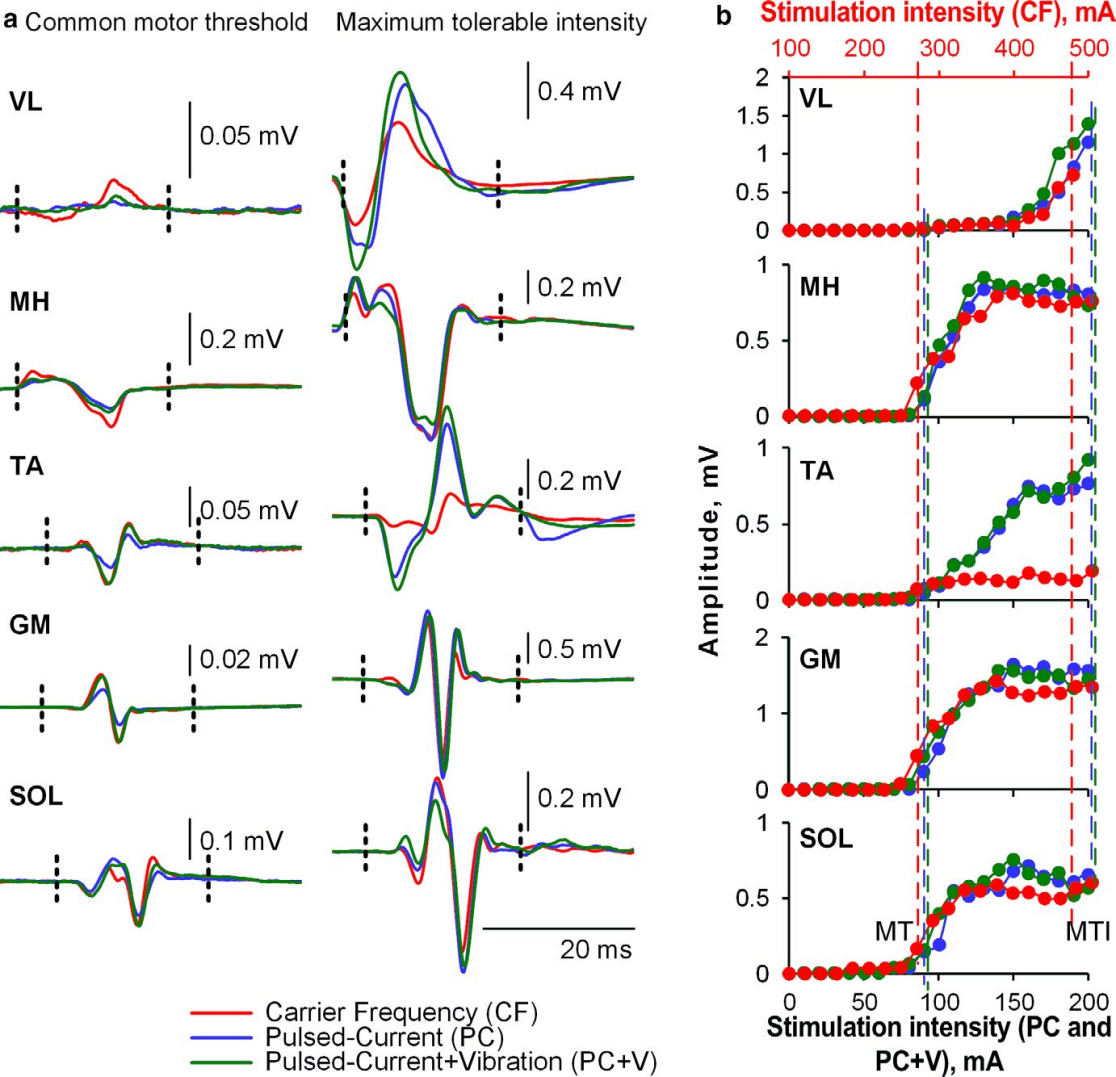
Examples of waveforms and recruitment curves obtained during Pulsed‐Current (PC), Pulsed‐Current + Vibration (PC + V), and Carrier Frequency (CF) stimulation paradigms in one representative participant (P5). (a) demonstrates the waveforms of spinally evoked motor potentials in different muscles recorded at stimulation intensity corresponding to the common motor threshold (left panel, 90 mA for PC and PC + V, and 260 mA for CF paradigms) as well as maximum tolerable intensity (right panel, 210 mA for PC and PC + V, and 480 mA for CF paradigms). Vertical dashed lines indicate the time windows in which the peak‐to‐peak amplitude of the responses was calculated. (b) demonstrates recruitment curves during each stimulation paradigm for each muscle: VL, vastus lateralis; MH, medial hamstring; TA, tibialis anterior; GM, medial gastrocnemius; SOL, lateral soleus. Note that the scale indicating stimulation intensity for CF is displayed in red at the top of the Figure. Vertical dashed lines indicate the stimulation intensities corresponding to the common motor threshold (MT) and maximum tolerable intensity (MTI)

EMG data were processed in LabChart (version 8.1.13 ADInstruments). Figure 2 outlines the methods used to analyze EMG data in this study. The spinally evoked motor potentials were calculated by measuring the responses’ peak‐to‐peak amplitude within a 20‐ms time window 5 ms after stimulation artifact, for each muscle. The magnitude of muscle responses was used to determine the motor threshold (see below).

The dependent variables analyzed for the Single‐Pulse Stimulation protocol were as follows: (a) sensation at the maximum tolerable intensity, defined using the NRS score; (b) the maximum tolerable intensity; (c) the stimulation intensity at motor threshold, that is the lowest stimulation intensity wherein a spinally evoked motor potential was visually detected for any muscle; and (d) the maximum tolerable intensity normalized to stimulation intensity at motor threshold (expressed as a percentage). The average peak‐to‐peak amplitude of the spinally evoked motor potential at motor threshold was 0.17 mV (*SD* = 0.128). Normalizing the maximum tolerable stimulation intensity to the stimulation intensity at motor threshold was used as a way to gauge the relationship between the sensation at maximum tolerance and motor response induced by TSS.

Maximum tolerable intensity, pain rating at the maximum tolerable intensity, motor threshold, and the maximum tolerable intensity normalized to the intensity at motor threshold, were submitted to separate one‐way repeated measures ANOVAs.

The dependent variables analyzed for the Continuous Stimulation protocol included: (a) the maximum tolerable intensity; (b) the maximum tolerable intensity in the Continuous Stimulation protocol normalized to the maximum tolerable intensity in Single‐Pulse Stimulation protocol (expressed as a percentage); and (c) the maximum tolerable intensity in the Continuous Stimulation protocol normalized to the motor threshold obtained in the Single‐Pulse protocol (expressed as a percentage). All variables were submitted to one‐way repeated measures ANOVAs.

### 2.6 Statistical analyses

All statistical analyses were performed using the Statistical Package for Social Scientists (SPSS: IBM Inc. version 20). For all repeated measures ANOVAs, alpha was set to *p* = .05. The Hyunh–Feldt correction was used to correct the degrees of freedom (corrected to 1 decimal place) when the assumption of sphericity was violated. Effect sizes are reported using partial eta squared (η_p_^2^) and post hoc tests for significant main effects were performed using Bonferroni corrected paired samples *t*‐tests.

## 3 RESULTS

For the Single‐Pulse Stimulation protocol, the analyses did not yield any significant main effect of stimulation paradigm for pain perception at max intensity *F* (2,18) = 1.1, *p* = .34, indicating that discomfort experienced at the maximum tolerable intensity was not different between any of the stimulation paradigms (*M* = 5.9, *SD* = 1.5 on the NRS rating scale). Conversely, the ANOVAs revealed significant main effects of stimulation paradigm for the maximum tolerable intensity *F* (1.2, 11.1) = 50.1, *p* < .001, *η*_p_^2^ = 0.85; stimulation intensity at motor threshold *F* (1.0, 9.3) = 59.0, *p* < .001, *η*_p_^2^ = 0.87, and the maximum tolerable intensity normalized to the intensity at motor threshold *F* (1.2, 11.1) = 4.7, *p* = .047, *η*_p_^2^ = 0.34.

**FIGURE 3:**
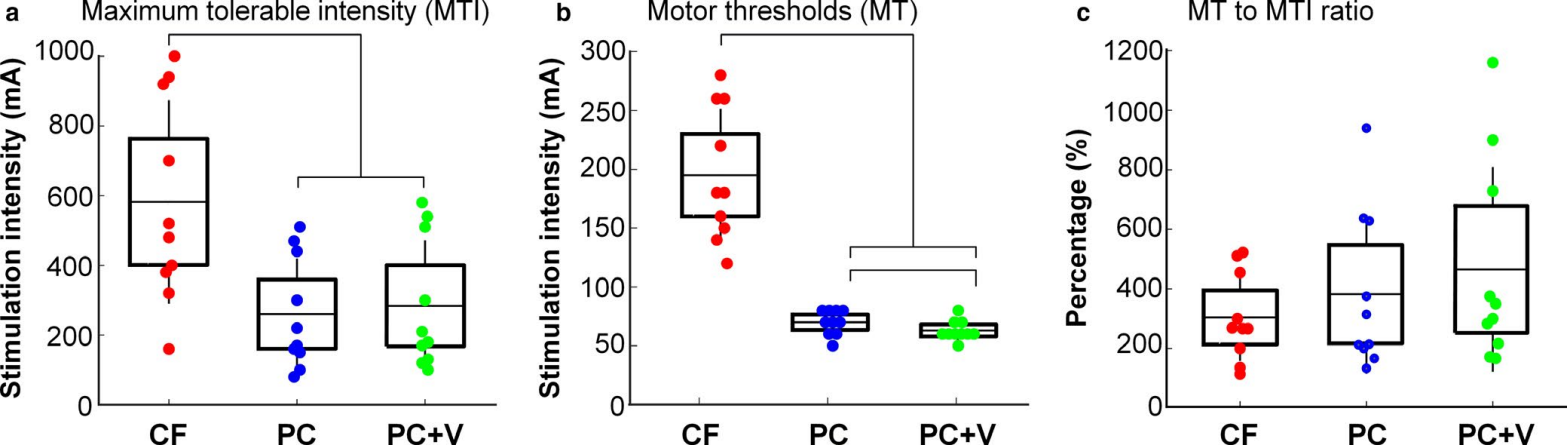
Summary of results for the Single‐Pulse Protocol. In each panel, the stimulation paradigm is labeled on the X‐axis: Carrier Frequency (CF), Pulsed‐Current (PC) and Pulsed‐Current + Vib (PC + V). Individual participant data are represented by colored circles, the box represents the mean and the standard error of the mean, and the whiskers show the standard deviation. (a) depicts the maximum tolerable intensity. (b) shows the motor thresholds. (c) shows the percentage of motor threshold to the maximum tolerable intensity

Post hoc tests (Bonferroni corrected alpha of 0.016) revealed that participants tolerated significantly higher stimulation intensities when exposed to the carrier frequency stimulation (*M* = 582 mA, *SD* = 291) compared to both the pulsed‐current (*M* = 260 mA, *SD* = 160; *p* < .001) and pulsed‐current + vib (*M* = 284 mA, *SD* = 188; *p* < .001) stimulation paradigms (Figure 3a). The differences between the pulsed‐current and pulsed‐current + vib stimulation paradigms were not statistically significant (*p* = .15). This result indicates that the participants tolerated the carrier frequency stimulation at higher intensities than either the pulsed‐current or the pulsed‐current + vib stimulation.

Similarly, post hoc tests revealed that the stimulation intensity required to attain motor threshold was significantly higher during the carrier frequency stimulation (*M* = 195 mA, *SD* = 56) than the pulsed‐current (*M* = 70 mA, *SD* = 10, *p* < .001) stimulation (Figure 3b). The tests also revealed that the stimulation intensity required to evoke a motor response during both carrier frequency and pulsed‐current simulations were significantly higher (carrier vs. pulsed‐current + vib: *p* < .001; pulsed‐current vs. pulsed‐current + vib, *p* = .009) than the pulsed‐current + vib stimulation (*M* = 63 mA, *SD* = 8). This result suggests that the addition of vibration may decrease the motor threshold.

Finally, for the percentage of motor threshold normalized to the maximum tolerable intensity, post hoc tests did not reveal any significant differences between the simulation paradigms: pulsed‐current (*M* = 380%, *SD* = 150); pulsed‐current + vib (*M* = 460%, *SD* = 340); carrier‐frequency stimulation (*M* = 300%, *SD* = 150) (Figure 3c). This result suggests that the differences in the absolute values of the maximum tolerable intensity are no longer present when the intensity required to obtain a motor response is considered.

**FIGURE 4:**
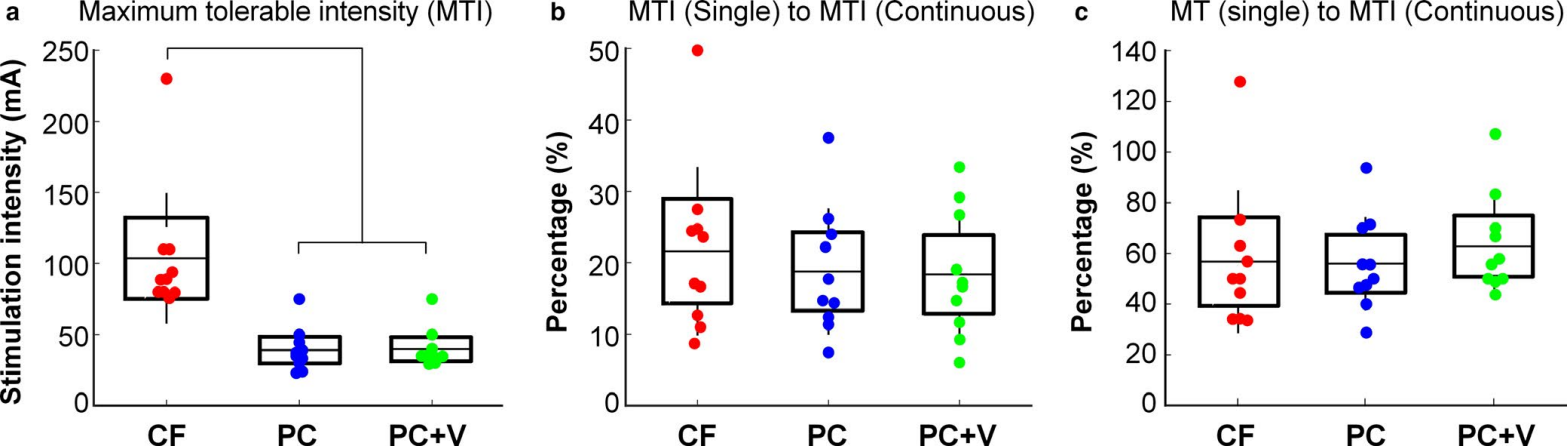
Summary of results for the Continuous Stimulation Protocol. In each panel, the stimulation paradigm is labeled on the X‐axis: Carrier Frequency (CF), Pulsed‐Current (PC) and Pulsed‐Current + Vib (PC + V). Individual participant data are represented by colored circles, the box represents the mean and the standard error of the mean, and the whiskers show the standard deviation. (a) the maximum tolerable intensity. (b) the relationship between the maximum tolerable intensity in the continuous protocol normalized to the maximum tolerable intensity in the single pulse protocol. (c) the maximum tolerable intensity in the continuous protocol normalized to motor threshold in the single pulse protocol

For the Continuous Stimulation protocol, only the analysis of the maximum tolerable intensity yielded a significant main effect of stimulus paradigm *F* (1.1, 9.5) = 35.9, *p* < .001, η_p_^2^ = 0.80. Post‐hoc tests (Bonferroni corrected alpha = 0.016) revealed that participants were able to tolerate stimulation with significantly higher intensities during the carrier frequency stimulation paradigm (*M* = 103 mA, *SD* = 46) than in both the pulsed‐current (*M* = 39 mA, *SD* = 15, *p* < .001) and pulsed‐current + vib stimulation paradigms (*M* = 39 mA, *SD* = 13, *p* < .001 see Figure 4a).

There were no differences between the stimulation paradigms when the maximum tolerable intensity obtained during the Continuous Stimulation protocol was normalized to the maximum tolerable obtained in the Single‐Pulse Stimulation *F* (1.4, 12.2) = 3.3, *p* = .09, (Figure 4b). Thus, the maximum tolerable intensity that a participant could tolerate during the Continuous Stimulation protocol was approximately 15% of the intensity that they tolerated during the Single‐Pulse Stimulation protocol regardless of the stimulation paradigm. Furthermore, similar to the results obtained in the Single‐Pulse Stimulation protocol, there were no differences between the stimulation paradigms when the maximum tolerable intensity was normalized to the motor threshold *F* (1.3, 11.3) = 0.9, *p* = .37, (Figure 4c). Overall, regardless of the stimulation paradigm, participants were able to tolerate stimulation intensities in the Continuous Stimulation protocol that were about 56% of their motor threshold.

With regard to the participants subjective reports of discomfort, it was found that participants reported the same reason for choosing to stop stimulation regardless of stimulation paradigm. The majority (e.g. *n* = 7 or 70%) of participants chose to stop stimulation because of discomfort associated with strong back muscle contractions, whereas only two participants stopped because of discomfort associated with skin irritation underneath the stimulation electrode. The remaining participant stopped because of strong abdominal contractions.

## 4 DISCUSSION

The purpose of this study was to examine the effects of two alternative stimulation paradigms on tolerance to TSS in neurologically intact individuals. Overall, we found that participants were able to tolerate higher intensities of TSS when stimulated using burst‐modulated biphasic waveforms (i.e., carrier frequency stimulation), as compared to unmodulated biphasic waveforms with or without vibration. Critically, when the maximum tolerable intensities for each stimulation paradigm were normalized to the stimulation intensities required to obtain a motor response, the relationship between the tolerance and motor thresholds were not different between the paradigms. The following discussion contextualizes these findings by drawing on the parallel literature of neuromuscular stimulation and examining the different physiological and physical characteristics of each stimulation paradigm.

Our finding that the carrier frequency resulted in less discomfort at higher stimulation intensities is supported by previous studies investigating neuromuscular stimulation (Ward et al., 2006). Similar to this study, many previous studies have also found that the advantages of the carrier frequency with regard to tolerance also come with deficits in performance (Bellew et al., 2018; Dantas, Vieira, Siqueira, Salvini, & Durigan, 2015; Ward & Chuen, 2009; Ward & Shkuratova, 2002). In particular, both Ward et al. (2006) and Dantas et al. (2015) showed that neuromuscular stimulation with the carrier frequency paradigm results in less muscular torque than stimulation paradigms using pulsed‐current. Similarly, a study by Bellew et al. (2018) found that the force elicited at a set level of discomfort was significantly lower for the carrier frequency stimulation compared to pulsed‐current stimulation.

One simple explanation for this phenomenon is that the amount of charge delivered by the burst‐modulated waveforms of 1 ms duration is lower than the amount of charge delivered unmodulated pulses with 1 ms duration (see Figure 1). Due to the 5 negative phases of 100 µs, the cathodic charge at the stimulating electrode was two times lower for the carrier stimulation paradigm as compared to both pulsed‐current paradigms. It is therefore not surprising that our results show that participants tolerated two times the intensity when exposed to the carrier frequency compared to paradigms with unmodulated pulses.

To elucidate the relationship between the participants’ tolerance to TSS and the induced motor response, the maximum tolerable stimulation intensity was normalized to the intensity to elicit spinally evoked motor potentials in lower limb muscles (i.e., the motor threshold). The motor threshold was chosen as a basic physiological parameter because it is indicative of the functional engagement of spinal sensorimotor networks. Neither the carrier frequency stimulation nor the addition of surface vibration affected the relationship between maximum tolerance and motor threshold. Although, participant's maximum tolerable intensities were two times higher for the carrier stimulation, the intensities required to elicit a muscle response were also about two times higher.

One can argue that the shorter pulses in the carrier frequency stimulation paradigm may not be optimal for spinally evoked motor potentials. For instance, it was previously shown that short pulse durations (0.05–0.4 ms) preferentially activates motor axons (Grill & Mortimer, 1996), whereas the use of longer pulse durations (0.5–1 ms) recruits relatively more sensory axons (Kiernan, Mogyoros, & Burke, 1996; Mogyoros, Kiernan, & Burke, 1996; Panizza, Nilsson, & Hallett, 1989). As such, it is possible that the carrier frequency did improve pain tolerance; but, motor thresholds in response to carrier stimulation were higher because the waveform is suboptimal for the activation of spinally evoked muscle responses. The idea that shorter pulses may not be optimal for spinally evoked motor responses is also supported by work of Lagerquist & Collins, 2008 demonstrating that wider pulse‐widths (1 ms) may be more suitable for increasing the reflex contribution to motor potentials.

Regardless of the exact mechanism, the ratio between tolerance and spinally evoked motor potentials has to be considered when choosing a stimulation paradigm for studies that require TSS‐induced motor activation for functional outcomes (e.g., for standing such as in Sayenko et al., 2019). On the other hand, previous studies have demonstrated the TSS can be used to facilitate other functions such as rhythmic motions (Gerasimenko et al., 2015), trunk stability (Rath et al., 2018), cardiovascular function (Phillips et al., 2018), and bladder function (Gad et al., 2018) in individuals with spinal cord injury, without *directly* inducing motor responses. These studies used the carrier frequency stimulation paradigm in an attempt to increase stimulation intensity and engage spinal interneural networks while reducing stimulation‐related pain. Thus, it is possible that stimulation using the carrier frequency may recruit additional interneuronal spinal circuitry during functional tasks, however, our data demonstrates that, in a cohort of neurologically intact individuals, there were no detectable differences in spinally evoked motor potentials. Future work should be done during these functional tasks and using different TSS waveforms and parameters to determine whether the carrier frequency is advantageous in engaging the interneuronal spinal, ascending, or descending neural networks.

Previous studies have also shown that there is a reduction in discomfort when vibration is applied to the same area affected by noxious stimuli. This was demonstrated for pain associated with radiant heat, itch, and electrical stimulation (Higgins et al., 1971; Melzack & Schecter, 1965; Melzack & Wall, 1965; Sullivan, 1968). In contrast to these studies, our results suggest that there is no benefit of vibration for continuous stimulation protocols when employing TSS. The finding that the addition of vibration and the carrier frequency did not significantly reduce discomfort should be considered with regard to the reasons as to why participants stopped continuous stimulation. Surprisingly, during both the conventional and the alternative stimulation paradigms, the majority of participants stopped stimulation because of discomfort associated with back muscle contractions, and not due to the skin sensation. These reports support the notion that factors other than the activation of skin nociceptors should be considered when using TSS paradigms aimed to minimize discomfort. The idea that factors other than skin nociceptors affect pain tolerance to TSS is also in agreement with the suggestions outlined by other experimental studies using TSS (e.g., Hofstoetter et al., 2018).

From a practical viewpoint, the present study demonstrated that participants were able to tolerate stimulation intensities up to 3.8 times higher than their motor threshold (380%) in the single‐pulse protocol (carrier frequency: 300%; pulsed‐current: 380%; pulsed‐current + vib: 460%). Similarly, participants, on average, were able to tolerate continuous stimulation at 56% of their motor threshold. These observations could prove useful for practitioners as a method to extrapolate the maximum tolerance during both single pulse and continuous TSS.

Lastly, it is worth noting that all evoked responses were recorded while participants were in a prone position. It has been previously demonstrated that body position influences which neural structures are recruited during TSS (Danner et al., 2016). Therefore, future work with participants in more functionally relevant positions such as standing or sitting, and/or using additional configurations of stimulating electrodes, may provide additional information. Additionally, the participant cohort within this study was neurologically intact. TSS in clinical settings will likely be applied to participants with preserved, but potentially altered sensory function such as incomplete SCI, stroke, or multiple sclerosis. Further studies on clinical populations would be useful for understanding the effect of TSS parameters and pain response in the presence of neurological injury.

## 5 CONCLUSION

In the present study, we compared two alternative stimulation paradigms to improve tolerance during TSS: a 5 kHz carrier frequency and unmodulated pulses with vibration, to conventional, unmodulated pulse stimulation. We hypothesized that both the carrier frequency and vibration stimulation paradigms would improve tolerance to TSS by reducing pain receptor activation. We normalized the results to the minimum intensity required to elicit a spinally evoked motor potential (i.e., the motor threshold), as an objective measure of the physiological response. Although participants tolerated higher intensities with the carrier frequency stimulation paradigm, there were no differences in the relationship between the maximum tolerable intensity and the intensity required to activate spinal sensorimotor circuits between the stimulation paradigms. Altogether these results suggest that neither of the alternative paradigms has the advantage of reducing pain while inducing a similar motor response, as compared with a conventional, unmodulated pulse configuration.

## CONFLICT OF INTEREST

None declared.
